# The Friendly Societies' Conferences

**Published:** 1913-05-24

**Authors:** 


### NATIONAL INSURANCE ACT DEPARTMENT.

### LIV.—The Friendly Societies' Conferences.

During Whit week, in accordance with custom, the great friendly societies held their annual conferences. These conferences were much more interesting this year than usual, not only to the delegates attending them and to the members of the societies directly concerned, but also to the general public. This wider interest is evident from the larger space allotted to the reports of the proceedings in the daily newspapers, and to the comments made thereon in most of the principal journals. The interest in the proceedings very naturally centred in the effect which the National Insurance Act has already had, and is still likely to have, on the societies. We have studied the published reports somewhat closely and propose to set forth what appear to us to be the main points in the reports, addresses, and speeches.

### Dissatisfaction with Regulations.

The most patent fact disclosed by all the speeches is that the National Insurance Act has not satisfied the friendly society officials. Though based largely on friendly society lines, and designed to provide special recognition to and benefits for those who had in the past made voluntary provision against sickness and disablement through friendly societies, the Act had failed to appeal to the officials of the societies because of its attendant multitudinous regulations and restrictions. Formerly as private and voluntary associations the friendly societies obtained the services of officers either gratuitously or at purely nominal salaries, the duties being chiefly carried out in spare time, and in consequence much of the work was inefficiently done, or done with a minimum of trouble and restriction. Everything has now had to be placed on a purely business footing, and any laxity which may previously have existed has had to give way to the more formal methods which alone can satisfy the requirements of a State scheme.

It is clear from the reports that even the most expert in friendly society administration have found no little difficulty in organising their societies under the altered conditions to meet the requirements of the new officials at the Insurance Commissions.

In this the officers of the friendly societies are, doubtless, deserving of sympathy, but that their lot is not likely to be lightened is clear from the remarks of Dr. Macnamara in his speech to the National Independent Order of Oddfellows. "Complaint had been made," he said, "by a delegate of the great amount of red-tape that was involved in the administration, but directly public money was involved it was in the public interest to see that it was most scrupulously administered. Public money must be administered subject to certain checks, which involved control which was new to them. He believed the machine would soon run smoothly." The officials will in course of time become accustomed to the regulations and requirements, and it is to be hoped that their emoluments will be increased to correspond to the greater complexity of their duties; but in the process it is to be feared that the societies will lose much of their former character, for, instead of being associations for mutual help which extended far beyond the mere provision of monetary benefits, they will merely be the channel for the distribution of State-aided grants payable only on the happening of certain well-defined contingencies.

### Medical Treatment of Old Members.

Quite apart from the personal complaints of officers of the societies there are evidences to show that the societies themselves have certain solid reasons to regret the effect of the Act on their voluntary operations. One of the direct effects of the Act has been to place an additional liability on the funds of the societies in respect of their old members. The dispute between the Chancellor of the Exchequer and the medical profession in regard to the terms that could be offered and accepted for the administration of medical benefit led to the resignation by the doctors of club practice as formerly in existence. So far as the members of the societies are insured persons under the Act the matter is of no consequence, for the doctors now serving on the panels are giving attendance to those members, and the cost is being provided out of the insurance contributions, supplemented by the special extra grant from the State. But in regard to the members who are not insured persons under the Act, and for whom no State aid is received, the societies have had to enter into fresh agreements with the doctors, and the cost of treatment has risen in some cases from 2s. 6d. to 8s. 6d. per member per annum. The persons chiefly affected are the old members who are no longer employed or employable, and it may be are permanently on the funds of the society as pensioners. These persons require more medical attendance than the healthy younger members, and the charge for attendance even at the figure of 8s. 6d. per annum can hardly be remunerative to the doctors, but the additional liability to the societies is one of considerable consequence.

### Heavy Sickness Claims and their Effect.

Another matter which is causing some anxiety to society officials in regard to the future of the societies is the much heavier rate of claim for sickness benefit experienced since the beginning of the year than in the corresponding period in former years. This high rate of claim has apparently not only affected the societies on their State side—or approved society section—but it has made itself manifest in the voluntary or private sides of the societies. It is possible, of course, that this adverse experience may be of a temporary character, but if it should prove to be a permanent feature of the societies under their altered conditions, or even if it should last for any considerable time, it is to be feared that it will portend the disappearance of at least some of the societies. Quite a large number of the branches of the societies, if not the societies themselves as a whole, have in the past shown deficiencies in their accounts on the basis of the actuarial valuations, and the reason that many of such branches have not already been wound up is that stringent precautions have been taken to ensure that only cases of *bona-fide* illness and of real necessity have been relieved from the society's funds. If now the salutary effect of the measures of self-reform are to be negatived the already weak financial position will speedily become more pronounced, until finally it will be evident that the commitments cannot be met, when of necessity the society will be dispersed and what little may remain of the funds will be distributed among the members. The prospect is a grave one, but it is to be hoped that measures will be taken immediately to suppress malingering, and that thus the friendly societies may be in no worse position financially than they were before the passing of the National Insurance Act.

Increase of Membership.

We have dealt first with some of the principal objections to the Act which were voiced at the conferences. On the other side there was much in the reports of the societies which was encouraging. In nearly all cases the larger societies report substantial increases in their voluntary membership, apart from the accession of members on the State section. This means that the Act has infused fresh life into the friendly society organisations, for it has to be admitted that for some years past the growth of the societies had been inconspicuous. Furthermore, consideration is apparently being given to plans for still further extending the scope of their organisation by undertaking life assurance on a larger scale and by the institution of juvenile branches or lodges from which to recruit members on the attainment of the age of sixteen, when insurance contributions become payable compulsorily under the State scheme. These are, of course, perfectly legitimate ways in which the societies may take steps to protect themselves in the future, but it is unfortunate that so much stress should be laid on the necessity for providing life assurance as a means of competing with the collecting societies and industrial assurance companies. It is urged by many impartial observers that the organisation of the friendly societies was such as to make it impossible that they alone should undertake the distribution of National Insurance benefits. Though in some districts the representatives of the approved societies formed in connection with the collecting societies and companies may have come into sharp contact with the old friendly society officials in canvassing for members, they have on the whole secured their members from their own former connections, and there is no justification for permanent jealousies on either side as to the success of their respective endeavours. The friendly societies, being numerically stronger than the approved societies of the other group, have, as a matter of fact, secured a larger number of insured persons. The approved societies formed under the auspices of the collecting societies and companies are, however, so large individually that special consideration must of necessity be given to them, though not at the expense of the smaller societies.

The desire for extension of membership found expression in the discussions of the Manchester Unity of Oddfellows, and as a result the directors were authorised to negotiate with the Insurance Commissioners for the taking over by their society, either alone or in conjunction with other approved friendly societies, of the administration of State benefits to all deposit contributors.

That the deposit contributor class is a particularly unfortunate one is admitted on all hands, and the position of the class must be brought under review before the end of next year, but the constitution of the class is so uncertain that it is a matter for grave doubt whether it would be to the advantage of any society to undertake the provision of the statutory benefits without obtaining some special guarantee from the Government. This guarantee could hardly be given in present circumstances without the offer being made equally to all societies, and we therefore view the proposal of the Manchester Unity with some misgivings as to its ultimate success, although it will certainly be watched with interest.

